# Optical coherence tomography angiography at the acute phase of optic disc edema

**DOI:** 10.1186/s40662-018-0109-y

**Published:** 2018-06-23

**Authors:** Marie-Bénédicte Rougier, Mélanie Le Goff, Jean-François Korobelnik

**Affiliations:** 1 Service d’Ophtalmologie, CHU de Bordeaux, Bordeaux, France; 2 Bordeaux Population Health Research Center, Team LEHA, University Bordeaux, INSERM, UMR 1219, F-33000 Bordeaux, France

**Keywords:** Optical coherence tomography angiography, Optic nerve head, Edema, NAION, Microvascular network, And morphology

## Abstract

**Background:**

The differential diagnosis of optic disc edema at the acute phase can be challenging. OCT angiography (OCTA) is a new technology allowing the visualization of the peripapillary vascular network and optic disc capillaries. The peripapillary network alterations of glaucoma and chronic non-arteritic anterior ischemic optic neuropathy (NAION) were reported. However, no OCTA studies on acute optic disc edema from various causes. The aim of this project was to use OCTA to demonstrate the vascular changes the optic nerve head of various types of optic disc edema at the acute phase.

**Methods:**

In this retrospective study, patients with non-arteritic anterior ischemic optic neuropathy (NAION), papillitis or papilledema were recruited. Each patient was imaged using the AngioPlex™ CIRRUS™ HD-OCT device(model 5000, Carl Zeiss Meditec, Inc., Dublin, USA) with a scanning area of 6 × 6 mm^2^ centered on the optic disc. A morphological analysis of the peripapillary network was performed. For some patients with unilateral optic disc edema, a quantitative analysis was performed using a swept-source OCT-A system (PLEX® Elite 9000, Carl Zeiss Meditec, Inc., Dublin, USA). Vessel perfusion density and flux index of the peripapillary area were calculated.

**Results:**

Eight eyes with NAION (4 patients), 12 eyes with papillitis (6 patients) and 25 eyes with papilledema (13 patients) were imaged. The apparent disappearance or moderate pattern alteration of the peripapillary capillary vessels were observed in patients with NAION or papillitis, respectively. For papilledema, the capillaries at the surface of the optic disc were dilated and tortuous, but no peripapillary network pattern changes were observed. The quantitative analysis did not show any difference of peripapillary network between NAION and healthy eyes. For papillitis, the flux index was higher in inflammatory eyes compared to the healthy eyes in average (*p* = 0.03).

**Conclusion:**

At the acute phase, the morphological analysis of OCT-A appeared to be more useful than the quantification analysis, facilitating the differentiation between the three kinds of ONH edema: ischemic, inflammatory and papilledema.

## Background

Using multimodal imaging, the diagnosis of optic disc edema is not difficult. However, finding the underlying causes could be challenging. Except for papilledema, which often involves both eyes and is easy to diagnose, the differentiation between ischemic and inflammatory optic neuropathies is not easy, and requires further ophthalmologic and non-ophthalmologic explorations. Ischemic and inflammatory optic neuropathies may present as various types of visual loss at any age. The fundus photo is rarely helpful in discriminating the causes, and the so-called typical altitudinal visual field defect encountered in ischemic optic neuropathy could be associated with papillitis. The fluorescein angiography is the key examination when the perfusion of the optic nerve head at the early phase could be caught. If there is a perfusion delay or hypoperfusion of the optic nerve, the most likely diagnosis is ischemic optic neuropathy. When the hypoperfusion is not caught early enough during fluorescein angiography, the dye leakage may hide the vasculature at the surface of the optic disc. In this case, the only assertion to be made is that there is optic disc edema. Furthermore, this imaging technique is invasive and cannot always be performed promptly since this device is not always accessible in small ophthalmological centers. The diagnosis of papillitis could be challenging when it is not associated with any intraocular inflammation or retro-ocular pain. The use of invasive investigations, such as lumbar puncture and cerebral imaging, are often necessary.

With OCT angiography (OCTA), the visualization of the retinal peripapillary and optic nerve head vessels is possible due to the detection of motion contrast from the blood flow [1]. With the automatic segmentation commonly provided by these commercially available systems, it is possible to detect the superficial vascular plexus at the level of the retinal nerve fiber layer (RNFL) and the ganglion cell layer. Spaide et al. [2] showed that unlike fluorescein angiography, OCTA allows visualization of the radial peripapillary capillaries (RPC). The capillary network appears radially distributed around the optic disc, with no vascular drop out in most eyes. The capillaries run along the nerve fibers, and the vascular density decreases centrifugally from the optic disc. This decrease of the capillaries correlates with the RNFL thickness. OCTA has been mainly used in glaucoma patients and demonstrated the decrease of vascular density and blood flow of the peripapillary network (see Akil et al. for review [3]). Optic disc edema, regardless of the cause, causes RPC pattern changes. Studies of optic disc edema of either miscellaneous optic neuropathies [1, 4] or non-arteritic anterior ischemic optic neuropathy (NAION) at the acute phase or after resolution of the edema [5–11], or optic neuritis [12–15] demonstrated modifications of either RPC or optic disk vascularization. However, the distinctive signs that could be sued to differentiate among various underlying causes have not been reported. The aim of this project was to describe various OCTA changes of the vasculature of the optic nerve head and RPC in the setting of various causes of optic disc edema in the acute phase. The study may help in the differential diagnoses of various kinds of optic disc edema at acute stages.

## Methods

### Patients

The study was approved by the institutional review board of the French Society of Ophthalmology and was conducted by the tenets of the Declaration of Helsinki*.* From September 1st, 2016 to October 1st, 2017, all consecutive patients diagnosed with acute optic disc edema related to either non-arteritic anterior ischemic optic neuropathy (NAION), papillitis or papilledema were recruited and imaged using an OCTA device. We defined acute optic disc edema when patients complained of a visual loss for less than ten days. For each category, the diagnosis was made based on standard clinical criteria.

All patients underwent a standardized ophthalmic examination including best corrected visual acuity, visual field, slit lamp examination, fundus photo, and B-scan OCT of the optic disc with the Enhanced Depth Imaging system to detect optic disc drusen (Spectralis®, Heidelberg Engineering, Heidelberg, Germany). Fluorescein angiography was performed on all patients with unilateral optic disc edema (Spectralis®, Heidelberg Engineering, Heidelberg, Germany). Furthermore, for NAION, a general examination and ESR/CRP dosage were performed to exclude arteritic ischemic optic neuropathy [16]. For papilledema, patients underwent a brain magnetic resonance imaging (MRI) and magnetic resonance venogram (MRV) to rule out papilledema secondary to intracranial lesion or cerebrovenous thrombosis. Lumbar puncture with opening pressure measurement was also performed. Patients were diagnosed with idiopathic intracranial hypertension following the Modified Dandy Criteria [17].

For papillitis, MRI and cerebrospinal fluid analysis were performed. As demonstrated previously, multiple sclerosis itself could induce modification of the vessel density [14, 15]. Therefore, we ruled out papillitis associated with multiple sclerosis or associated with autoantibodies (anti-NMO and anti-MOG). Papillitis was diagnosed as optic disc edema associated with the usual inflammatory criteria (quick onset of visual loss, retro-ocular pain, and slight intraocular inflammation) and the absence of early hypoperfusion of the optic disc on the fluorescein angiography.

Finally, we excluded individuals with other optic neuropathy, such as glaucoma, optic disc drusen and amiodarone optic neuropathy, which may affect OCTA analysis. We also excluded patients for whom OCTA images were of poor quality.

### OCTA acquisition

Our study was conducted in two parts. The first one consisted of a morphological analysis of the RPC of all eyes with optic disc edema. The second part was dedicated to quantitative analysis that only included the unilateral optic disc edemas, i.e., NAION and papillitis. For this quantitative examination, each affected eye was compared to the contralateral healthy eye.

For morphological analysis, each patient underwent OCTA on both eyes using the AngioPlex™ CIRRUS™ HD-OCT device(model 5000, Carl Zeiss Meditec, Inc., Dublin, USA) with a scanning area of 6 × 6 mm^2^ centered on the optic disc. A fully-automated retinal layer segmentation algorithm was applied to select a superficial slab that included the RNFL and the ganglion cell layer. A morphological analysis of the peripapillary network was performed by a skilled physician.

For quantitative analysis, and only for patients with unilateral optic disc edema, a swept source OCT-A using the PLEX® Elite 9000 device (Carl Zeiss Meditec, Inc., Dublin, USA) was performed on both eyes. OCTA 6 × 6 mm^2^ scans centered on the optic nerve head (ONH) were acquired. A fully-automated retinal layer segmentation algorithm was applied to three-dimensional structural OCT data to segment the inner limiting member (ILM) and the outer boundary of RNFL for peripapillary scans. The segmentation results were then applied to OCT-A flow intensity data to obtain vascular images. Maximum projection analyses of the flow intensity were performed to generate the radial peripapillary capillaries (RPC) plexus from ILM to RNFL for peripapillary scans.

Once acquired, all raw data were exported and sent to the Carl Zeiss Meditec Inc. Ophthalmic Diagnostics R&D, which analyzed the data with a prototype algorithm and provided the vessel perfusion density (VPD) and flux index (FI) values. The quantification process has already been described [18]. The VPD is defined as the total area of perfused vasculature per unit area in a region of interest (ROI). It is calculated by averaging the binary vessel image within the desired ROI. The FI is defined as the total weighted area of perfused vasculature per unit area in an ROI. The weight is the flow intensity corresponding to each pixel. Essentially, the flux index is calculated by averaging the intensity of all vessel pixels divided by total vessel pixels within the ROI.

**Fig. 1:**
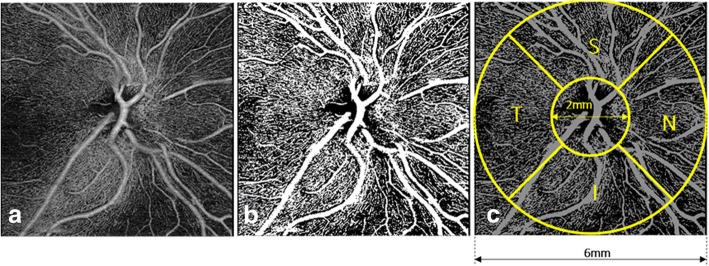
Data processing algorithm overview. (**a**) RPC (from ILM to RNFL) angiography slab from angiography 6 × 6 mm^2^ scan. (**b**) Vessel binary image where the large retinal vessels have been removed. (**c**) Vessel density and flux index metrics calculation. VD and FI are calculated within the red annulus with inner and outer diameters of 2 mm and 6 mm, respectively. Values are reported for four superior, nasal, inferior, and temporal quadrants and the entire annulus. RPC, retinal peripapillary capillaries; ILM, internal limiting membrane; RNFL, retinal nerve fiber layer; VD, vessel density; FI, flux index

The ROI is a ring-shaped pattern centered on the ONH (inner circle two mm-diameter, outer circle six mm-diameter) superimposed onto the peripapillary OCT-A scan. The areas outside of the outer circle and within the inner circle (including the ONH) were colored out black, and were excluded from the analysis; thus, only the parts of the image included in the ROI was used to calculate the RPC-VPD and RCP-FI. Finally, the large vessels were excluded from the image, to calculate only the capillary density. The VD and FI were calculated in 4 quadrants (Superior, Nasal, Inferior and Temporal) and averaged accordingly (Fig. 1).

Each eye was compared with the unaffected fellow eye to alleviate the abnormalities due to the patient’s medical background, which may have had consequences on both eyes. By doing so, we emphasized the changes that can only be due to the acute event.

The Wilcoxon test for matched samples was used. A *p*-value less than 0.05 was considered statistically significant.

## Results

Eight eyes (4 patients) with NAION, 12 eyes (6 patients) with papillitis and 25 eyes (13 patients)with papilledema were included in the present study.

### Morphological analysis

**Fig. 2:**
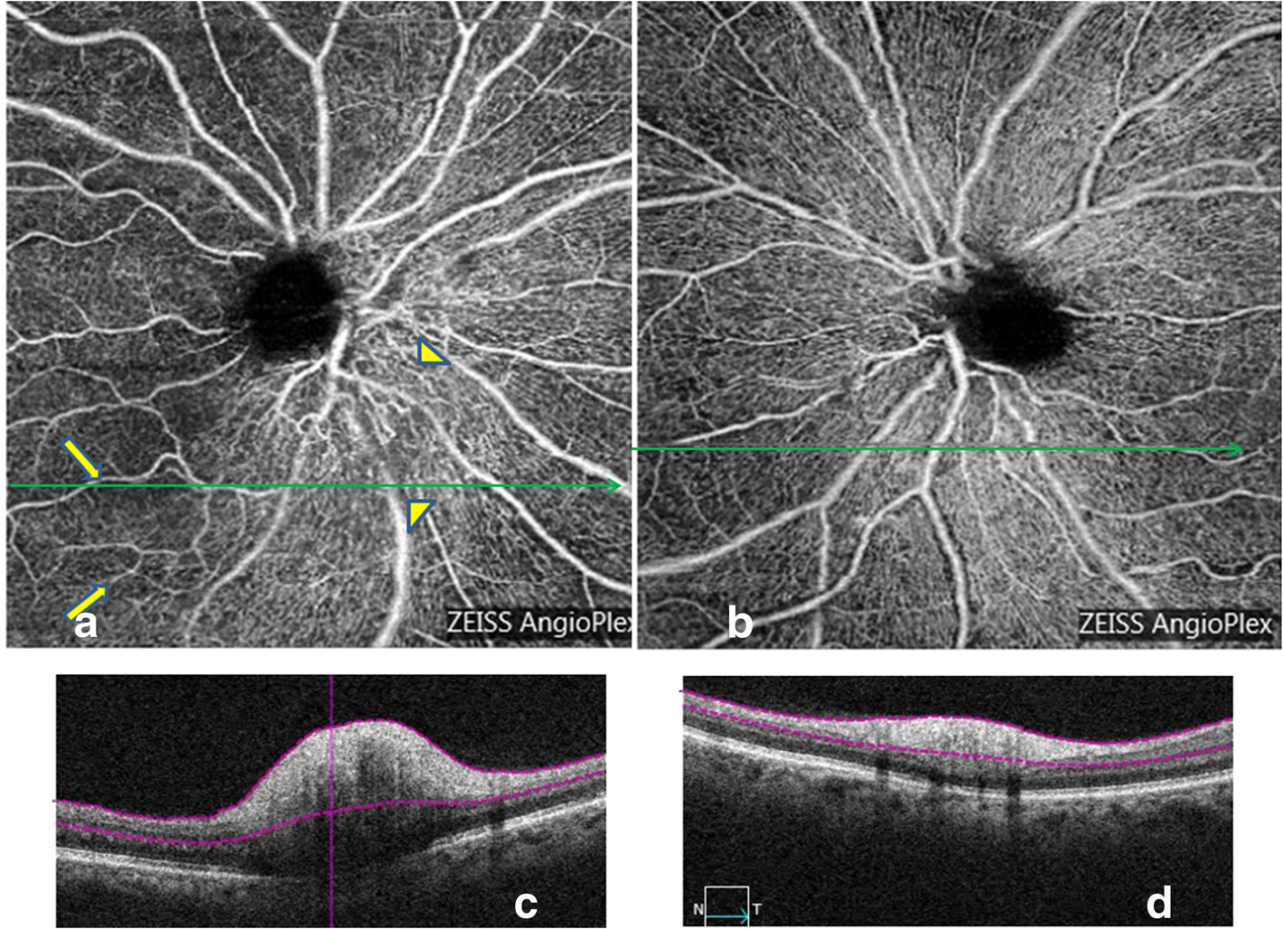
A 65 years old woman with an acute visual loss of her right eye related to NAION**.** Superficial OCT-A (Angioplex©) of her right eye (**a**) shows on the nasal side of the optic disc a dilation of the capillaries, which appear tortuous (arrowheads). On the temporal side, there are some dark areas corresponding to a vascular drop out (arrows). The B-scan inferior to the optic disc shows that there is no edema in the vascular dropout area, and thus no shadowing artifact (**c**). In the healthy eye (**b** and **d**), the peripapillary network appears regular with no vascular dropout. Green arrows within the OCT-A images indicate the location and direction of the B-Scans

Ischemic optic neuropathy: in all NAION eyes, the peripapillary network was less visible, especially around the edema area (Fig. 2). There was some vascular drop out (arrows), displayed by dark areas. In some eyes, vascular tortuosity at the surface of the optic disc was visible (arrowheads). The global aspect was a severe disappearance of the peripapillary regular pattern.

**Fig. 3:**
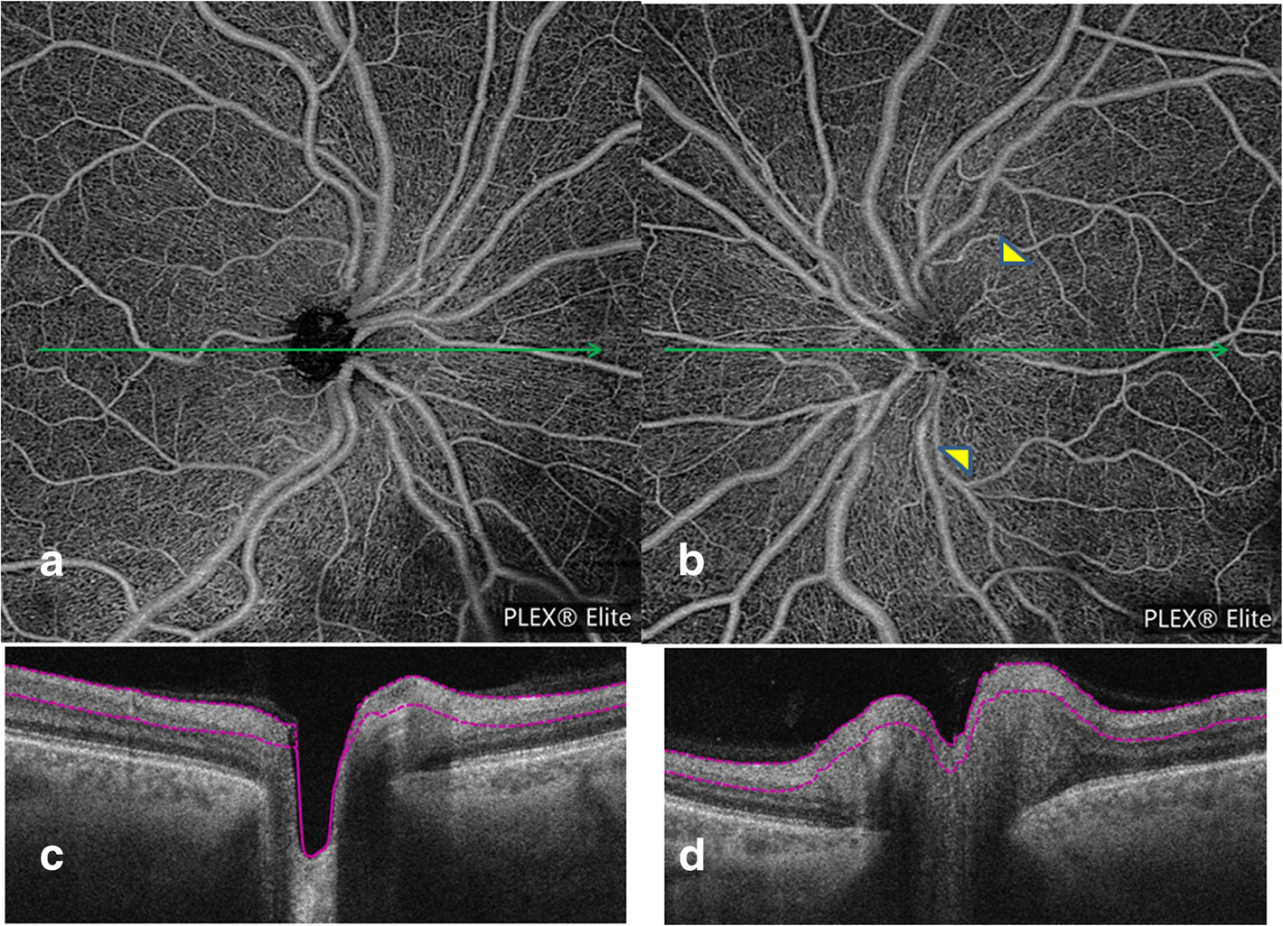
52 years old male presenting a papillitis on the left eye related to syphilis infection. Superficial OCT-A (Plexelite©) on the left eye (**b**) is almost normal, and the superficial optic disc vessels are slightly dilated (arrowheads). There is no vascular dropout or vascular tortuosity. When compared to the healthy eye (**a**), the peripapillary network appears regular. OCT-B scans (**c** and **d**) corresponding to a and b

Papillitis: in inflammatory optic disc edema, no vascular drop out was present. When the ONH edema was significant, the capillaries sometimes disappeared in the edema, but they were visible above the edematous area. More often, the radial distribution of the peripapillary capillaries remained preserved, and some vascular dilation could be seen in some eyes (Fig. 3). The global aspect was a moderate alteration of the peripapillary regular pattern.

**Fig. 4:**
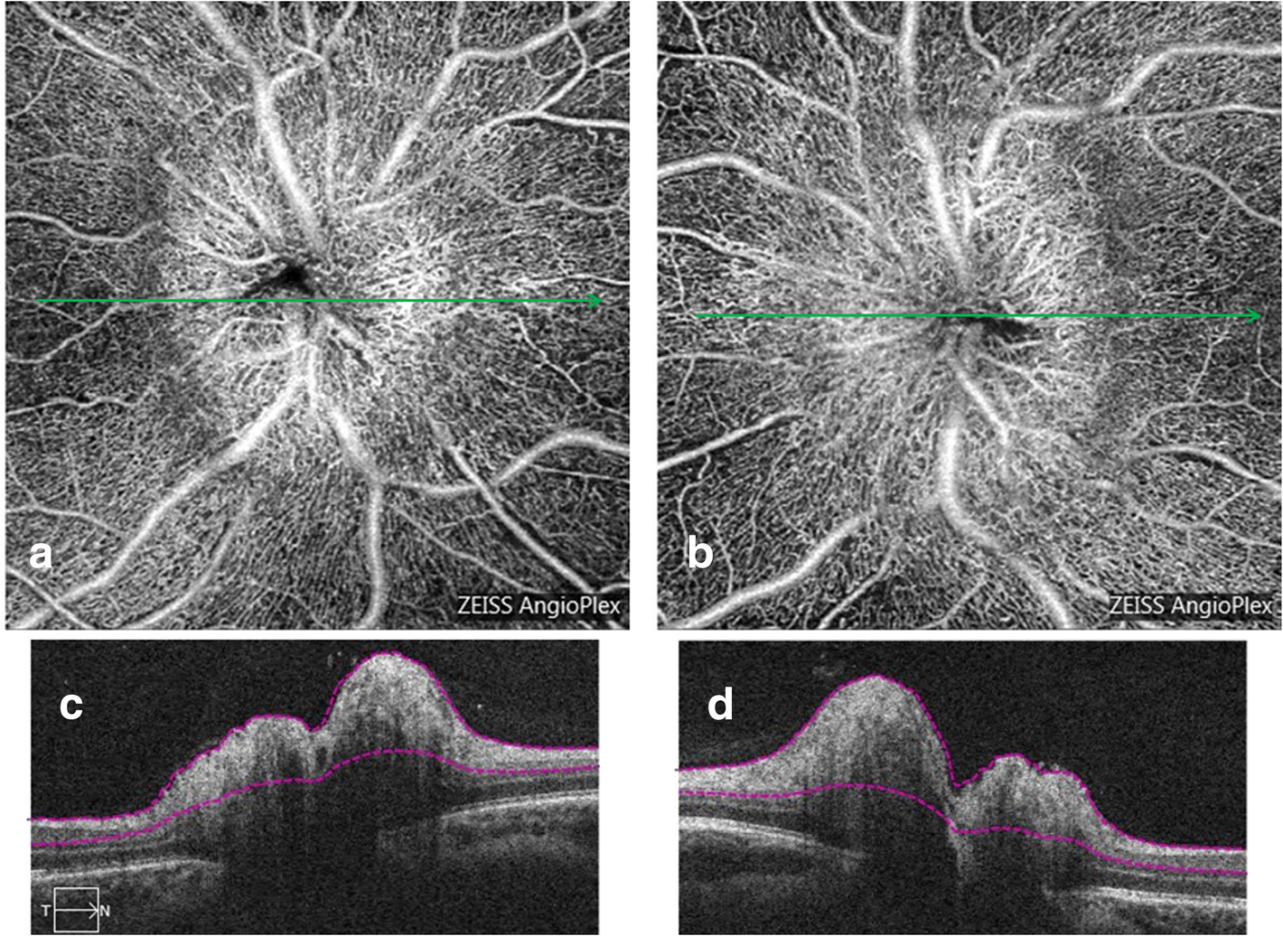
A 25 years old female presenting bilateral papilledema related to idiopathic intracranial hypertension. Superficial OCT-A of both eyes (**a** and **b**) acquired with Angioplex© demonstrating a dilation of the superficial optic disc vessels, which are tortuous and dilated. The global aspect of the optic disc looks like a tangled ball of vessels (arrowheads). OCT-B scans (**c** and **d**) corresponding to a and b

Papilledema: in 12 patients, the papilledema was bilateral. The 13th patient presented unusual unilateral papilledema, but the opening pressure measured during the lumbar puncture confirmed the diagnosis of idiopathic intracranial hypertension. In these eyes, the radial peripapillary network was unchanged. The capillaries at the surface of the optic nerve head were dilated and tortuous like a tangled ball of vessels (Fig. 4, arrowheads). There was no vascular dropout. The global appearance was of a bushy aspect.

### Quantitative analysis

Only 4 NAION and six papillitis eyes were quantified (Table 1). For NAION, no differences either for vessel perfusion density and capillary flux index were found compared with unaffected contralateral eyes. For papillitis, the FI was higher in inflammatory eyes when compared with the healthy eyes on average (*p = 0.03*). There was no difference for VPD. As the quadrant values are components of the global value, we did not consider the results of the different quadrants.

**Table 1:** Peripapillary Flux Index (FI) and Vessel Perfusion Density (VPD) mean variations and *p* Values in NAION eyes and papillitis

	NAION (N= 4)		Papillitis (N= 6)	
	Mean variation (SD)	*p* Value	Mean variation (SD)	*p* Value
FI global	0.0027 (0.0104)	0.8750	0.0243 (0.0229)	0.0313
FI temporal	0.0179 (0.0147)	N/A	0.0258 (0.0258)	N/A
FI superior	−0.0121 (0.0256)	N/A	0.0277 (0.0188)	N/A
FI nasal	0.0235 (0.0147)	N/A	0.0299 (0.0204)	N/A
FI inferior	−0.0244 (0.0355)	N/A	0.0120 (0.0277)	N/A
VPD global	−0.0126 (0.0289)	0.6250	−0.0136 (0.0211)	0.2188
VPD temporal	−0.0072 (0.0285)	N/A	0.0030 (0.0425)	N/A
VPD superior	−0.0356 (0.0273)	N/A	−0.0131 (0.0618)	N/A
VPD nasal	0.0050 (0.0404)	N/A	0.0002 (0.0264)	N/A
VPD inferior	−0.0171 (0.0734)	N/A	−0.0447 (0.0284)	N/A

The mean variation corresponds to the difference between involved eyes and unaffected fellow eyes

## Discussion

Despite many publications describing OCTA features of optic neuropathies, to the best of our knowledge, this is the first study looking at OCTA at the acute phase of optic nerve head edema to explore salient features that may guide the diagnostic investigation. Except for bilateral ONH edema, which is most often related to papilledema, finding the underlying cause of a unilateral ONH edema can be difficult. Fluorescein angiography can be of great help only if the early frames have detected a delay in the ONH filling, leading to the diagnosis of a NAION. Otherwise, the dye leakage prevents from the visualization of the optic disc vascularization. Patient background, past medical history, associated symptoms and visual field are other parameters that may guide the etiologic diagnosis. Nevertheless, in some cases, differentiating a NAION from a papillitis remains equivocal. In this case, an additional quick and safe diagnostic clue could be helpful.

It is necessary to state that in this study the main issue was the acquisition procedure with NAION eyes. Many eyes were excluded because of the poor quality of the images related to eye movement artifacts due to low visual acuity. This is the reason why even NAION is a common optic nerve disease, fewer patients have been included.

In the acute phase of NAION, we found an alteration of the RPC visualization associated with some vascular dropout and disappearance of the peripapillary regular pattern. These features have been described previously, and our observations confirm the blood flow impairment associated with NAION [1, 5–8]. However, unlike what reported in previous studies, we did not find any difference in vessel density nor the flux index between NAION eyes and unaffected fellow eyes [6, 7, 9]. This could be due to our small sample size. Or it could be because we compared NAION eyes with the healthy fellow ones while in other studies, the comparison was made with a group of normal control eyes [6]. It is known that NAION patients are more prone to have diabetes, hypertension, and sleep apnea, which could induce a retinal vascular dropout as described previously [19]. By comparing the NAION eye to the contralateral one, we tried to diminish the abnormalities due to systemic diseases or medication, and hence detected changes only due to the acute ischemic event. However, these modifications may not be detectable in the acute phase. In fact, we speculate that the VD and FI are not modified at the early phase but only at the chronic stage. It is known that NAION is the consequence of short posterior ciliary artery occlusion and that RNFL atrophy occurs consecutively. The reduction of nerve fibers induces an autoregulatory decrease of retinal blood flow due to lower metabolic needs. This blood flow impairment could thus be quantified through VD and FI reduction [6, 9–11]. The same mechanism has been suggested in glaucoma and optic neuritis [20, 21]. Therefore, at the acute phase of a NAION, OCTA morphological analysis might be more useful than the quantification of VD or FI.

Concerning papillitis, this study is the first one that examined RPC in the acute phase. The main differentiating factor of papillitis from NAION was no vascular dropout. Even in the case of significant edema, which may hide the peripapillary capillaries, they re-appear beyond the edema. In the present study, vascular dilation generally encountered in inflammatory processes was observed by quantitative studies. These quantitative results are promising and may represent a useful tool for the diagnosis of papillitis.

Lastly, we explored the morphological modifications of ONH in papilledema. The OCTA was performed when the patient was diagnosed with optic nerve head edema, but we could not obtain information regarding the duration of the edema. If referring to the onset of headache, the papilledema could have happened several months before it was detected. The OCTA demonstrated a specific presentation of the capillaries at the surface of the optic nerve head, which displayed a tangled ball of vessels. There was no modification at the level of the RPC. The relationship between intracranial hypertension and the curly dilation of the capillaries is not established, which warrants further investigation.

## Conclusions

In summary, OCT-A is a safe imaging modality that helps in the differentiation between the three kinds of ONH edema: ischemic, inflammatory and papilledema. At the acute phase, the morphological analysis appears to be more convenient than quantification because the edema itself may induce artifacts during the image acquisition affecting the quantification process. However, if it is confirmed by a larger series, the quantification could be an added tool for the diagnosis of the inflammatory swollen optic disc.

## References

[1] Ghasemi Falavarjani K, Tian JJ, Akil H, Garcia GA, Sadda SR, Sadun AA (2016). Swept-source optical coherence tomography angiography of the optic disk in optic neuropathy. Retina.

[2] Spaide RF, Klancnik JM Jr, Cooney MJ. Retinal vascular layers imaged by fluorescein angiography and optical coherence tomography angiography. JAMA Ophthalmol. 2015;133:45–50. 10.1001/jamaophthalmol.2014.3616 25317632

[3] Akil H, Falavarjani KG, Sadda SR, Sadun AA. Optical coherence tomography angiography of the optic disc; an overview. J Ophthalmic Vis Res. 2017;12:98–105. 10.4103/2008-322X.200162 PMC5340069 28299012

[4] Chen JJ, AbouChehade JE, Iezzi R, Leavitt JA, Kardon RH (2017). Optical coherence angiographic demonstration of retinal changes from chronic optic neuropathies. Neuroophthalmology.

[5] Balducci N, Morara M, Veronese C, Barboni P, Casadei NL, Savini G (2017). Optical coherence tomography angiography in acute arteritic and non-arteritic anterior ischemic optic neuropathy. Graefes Arch Clin Exp Ophthalmol.

[6] Song Y, Min JY, Mao L, Gong YY. Microvasculature dropout detected by the optical coherence tomography angiography in nonarteritic anterior ischemic optic neuropathy. Lasers Surg Med. 2018;50:194–201. 10.1002/lsm.22712 28986994

[7] Sharma S, Ang M, Najjar RP, Sng C, Cheung CY, Rukmini AV (2017). Optical coherence tomography angiography in acute non-arteritic anterior ischaemic optic neuropathy. Br J Ophthalmol.

[8] Rougier MB, Delyfer MN, Korobelnik JF (2017). OCT angiography of acute non-arteritic anterior ischemic optic neuropathy. J Fr Ophthalmol.

[9] Hata M, Oishi A, Muraoka Y, Miyamoto K, Kawai K, Yokota S, et al. Structural and functional analyses in nonarteritic anterior ischemic optic neuropathy: optical coherence tomography angiography study. J Neuroophthalmol. 2017;37:140–8. 10.1097/WNO.0000000000000470 27984351

[10] Higashiyama T, Ichiyama Y, Muraki S, Nishida Y, Ohji M. Optical coherence tomography angiography in a patient with optic atrophy after non-arteritic anterior ischaemic optic neuropathy. Neuroophthalmology. 2016;40:146–9. 10.3109/01658107.2016.1162174 PMC5123121 27928400

[11] Liu CH, Kao LY, Sun MH, Wu WC, Chen HS. Retinal vessel density in optical coherence tomography angiography in optic atrophy after nonarteritic anterior ischemic optic neuropathy. J Ophthalmol. 2017;2017:9632647. 10.1155/2017/9632647 PMC5337785 28316838

[12] Higashiyama T, Nishida Y, Ohji M (2017). Optical coherence tomography angiography in eyes with good visual acuity recovery after treatment for optic neuritis. PLoS One.

[13] Spain RI, Liu L, Zhang X, Jia Y, Tan O, Bourdette D (2018). Optical coherence tomography angiography enhances the detection of optic nerve damage in multiple sclerosis. Br J Ophthalmol.

[14] Lanzillo R, Cennamo G, Criscuolo C, Carotenuto A, Velotti N, Sparnelli F, et al. Optical coherence tomography angiography retinal vascular network assessment in multiple sclerosis. Mult Scler. 2017:1352458517729463. 10.1177/1352458517729463. 10.1177/1352458517729463 28933233

[15] Feucht N, Maier M, Lepennetier G, Pettenkofer M, Wetzlmair C, Daltrozzo T, et al. Optical coherence tomography angiography indicates associations of the retinal vascular network and disease activity in multiple sclerosis. Mult Scler. 2018:1352458517750009. 10.1177/1352458517750009. 10.1177/1352458517750009 29303033

[16] Characteristics of Patients With Nonarteritic Anterior Ischemic Optic Neuropathy Eligible for the Ischemic Optic Neuropathy Decompression Trial (1996). Arch Ophthalmol.

[17] Friedman DI, Jacobson DM (2002). Diagnostic criteria for idiopathic intracranial hypertension. Neurology.

[18] Chen CL, Zhang A, Bojikian KD, Wen JC, Zhang Q, Xin C, et al. Peripapillary Retinal Nerve Fiber Layer Vascular Microcirculation in Glaucoma Using Optical Coherence Tomography–Based Microangiography. Invest Opthalmol Vis Sci. 2016;57:475–85. 10.1167/iovs.15-18909 PMC4968914 27442341

[19] Ting DSW, Tan GSW, Agrawal R, Yanagi Y, Sie NM, Wong CW (2017). Optical coherence tomographic angiography in type 2 diabetes and diabetic retinopathy. JAMA Ophthalmol.

[20] Liu CH, Wu WC, Sun MH, Kao LY, Lee YS, Chen HS. Comparison of the retinal microvascular density between open angle glaucoma and nonarteritic anterior ischemic optic neuropathy. Invest Opthalmol Vis Sci. 2017;58:3350–6. 10.1167/iovs.17-22021 28687846

[21] Wang X, Jia Y, Spain R, Potsaid B, Liu JJ, Baumann B (2014). Optical coherence tomography angiography of optic nerve head and parafovea in multiple sclerosis. Br J Ophthalmol.

